# Comparison of Obturation Quality between Calcium Silicate-Based Sealers and Resin-Based Sealers for Endodontic Re-treatment

**DOI:** 10.3390/ma15010072

**Published:** 2021-12-23

**Authors:** Hye-Ryeon Jin, Young-Eun Jang, Yemi Kim

**Affiliations:** Department of Conservative Dentistry, College of Medicine, Ewha Womans University, Seoul 07986, Korea; jhr8141@naver.com (H.-R.J.); jang@ewha.ac.kr (Y.-E.J.)

**Keywords:** calcium silicate, endodontic Re-treatment, endodontic sealer, filling quality, micro-computed tomography, retrievability

## Abstract

Background: The objective of this micro-computed tomography (micro-CT)-based study was to compare the filling quality of endodontic treatment and endodontic Re-treatment between two sealers with matched obturation techniques: calcium silicate-based sealer (Endoseal TCS) with a single-cone technique (SCT) and resin-based sealer (AH Plus) with a continuous wave technique (CWT). Methods: Forty maxillary premolars were selected and assigned into four groups, according to the obturation methods of the first endodontic treatment and Re-treatment (*n* = 10). The AP/AP group was first treated with AH Plus/CWT, then re-treated with AH Plus/CWT. The AP/ET group was first treated with AH Plus/CWT, then re-treated with Endoseal TCS/SCT. The ET/AP group was first treated with Endoseal TCS/SCT, then re-treated with AH Plus/CWT, and the ET/ET group was first treated with Endoseal TCS/SCT, then re-treated with Endoseal TCS/SCT. The specimens were scanned using micro-CT at three time points: after the first endodontic treatment, after gutta-percha (GP) cone removal, and after Re-treatment. The void volume of root canal obturation and the volume of the remaining filling materials were calculated. Data were analyzed using Student’s *t*-tests and ANOVA. Results: The Endoseal TCS groups (ET/AP and ET/ET) showed a lower percentage of voids than the AH plus groups (AP/AP and AP/ET) on the whole canal and the apical third, after first obturation (*p* < 0.05). The AH plus group showed significantly fewer remnants than the Endoseal TCS group after GP removal (*p* < 0.05). Re-treated canals and initially treated canals had similar void volumes (*p* > 0.05). There was no significant difference in void volume after Re-treatment, regardless of whether the same or different sealers were used for the first treatment and Re-treatment (*p* < 0.05). Conclusions: Endoseal TCS sealer and AH Plus sealer had a similar Re-treatment efficacy, regardless of which sealer was used in the previous treatment.

## 1. Introduction

In recent years, calcium silicate-based sealers such as Endoseal MTA (Maruchi, Wonju, Korea), MTA Fillapex (Angelus, Londrina, Brazil), and Endosequence BC (Brassler USA, Savannah, GA, USA) have been introduced in endodontics. As these sealers are composed of mineral trioxide aggregate (MTA)-derived materials, they have biocompatibility [1,2,3], antibacterial effects [4,5], and a superior sealing ability [6,7,8]. Endoseal TCS (Maruchi) is a recently developed calcium silicate-based sealer intended to replace Endoseal MTA; it is composed of tricalcium silicate, phyllosilicate mineral, zirconium oxide, and dimethyl sulfoxide [9]. Endoseal TCS is a pure tricalcium silicate-based sealer, whereas Endoseal MTA is a Pozzolan cement-based sealer [9,10]. However, there have been few studies of the physicochemical properties of Endoseal TCS.

The object of canal obturation is the achievement of a fluid-tight seal that prevents intracanal recontamination [11]. Previous studies have shown that calcium silicate-based sealers have a similar obturation quality and intracanal void percentage, compared to AH Plus [12,13,14]. During endodontic Re-treatment, it is important to remove filling materials from the initial treatment. Several previous studies using micro-computed tomography analysis (micro-CT) have shown that it is impossible to completely remove filling materials, regardless of the obturation technique [15,16,17]. The residues could serve as sources of bacteria that cause root canal re-infection, and interfere with physical obturation during Re-treatment. To our knowledge, there have been no studies concerning whether calcium silicate-based sealers and resin-based sealers have similar obturation ability during initial treatment or Re-treatment.

Therefore, the objective of this micro-CT-based study was to compare the filling quality on first endodontic treatment and subsequent Re-treatment between two sealers with a matched obturation technique: calcium silicate-based sealer (Endoseal TCS) with a single-cone technique (SCT) and resin-based sealer (AH Plus) with a continuous wave technique (CWT). The null hypothesis was that there was no significant difference between the calcium silicate-based sealer and the resin-based sealer concerning the obturation quality in endodontic Re-treatment.

## 2. Materials and Methods

### 2.1. Sample Preparation

The study protocol was approved by the Institutional Review Board of the Ewha Womans University Hospital (EUMC 2021-02-008). In total, 40 extracted single-root human maxillary premolars were obtained from patients who had provided informed consent. Teeth were immersed in 10% formalin solution for 2 weeks to disinfect both the internal and external structures of the teeth. Initial periapical radiography was performed, and the teeth with fully formed apices with an oval-shaped canal and a single apical foramen were included. Teeth with open apex, signs of cracks, perforation or caries, or with endodontic treatment were excluded. Included teeth with oval-shaped canals were prepared like 2 canals. The crown portion of each tooth was transversely sectioned on the cementoenamel junction using a diamond saw.

After coronal access had been prepared, a size 10 K-file (Dentsply Maillefer, Ballaigues, Switzerland) was placed into the canal, and patency was achieved. When the tip of the file was visible at the apical foramen, the working length was established by subtracting 0.5 mm from the measured length. Then, both canals were instrumented with the ProTaper Universal Ni-Ti rotary system (Dentsply Sirona, Ballaigues, Switzerland) up to the F3 file, using 2.5% sodium hypochlorite between each file. Subsequently, the canals were soaked with 2 mL 17% ethylenediaminetetraacetic acid for 2 min, rinsed with 5 mL distilled water, and then soaked with 5 mL 5.25% sodium hypochlorite for 5 min. Finally, the canals were dried with F3 paper points (Dentsply Sirona).

### 2.2. Root Canal Obturation

The teeth were randomly assigned into the following 4 groups (*n* = 10) according to the obturation methods of first endodontic treatment and Re-treatment. AP/AP group: first treatment with AH Plus + CWT and Re-treatment with AH Plus + CWT. AP/ET group: first treatment with AH Plus + CWT and Re-treatment with Endoseal TCS + SCT. ET/AP group: first treatment with Endoseal TCS + SCT and Re-treatment with AH Plus + CWT. ET/ET group: first treatment with Endoseal TCS + SCT and Re-treatment with Endoseal TCS + SCT.

In the groups first treated with AH Plus (AP/AP and AP/ET), each root canal was obturated with an F3 gutta-percha (GP) cone (Dentsply Maillefer) and AH Plus using CWT. AH Plus pastes were mixed in accordance with the manufacturer’s instructions; the GP cones were smeared with sealer and placed into the canal. Then, down packing with a System B device (SybronEndo, Orange, CA, USA) was performed within 3–5 mm of the working length, and backfilling was accomplished using the SuperEndo-Beta (B&L Biotech USA, Bala Cynwyd, Montgomery County, PA, USA).

In the groups first treated with Endoseal TCS (ET/AP and ET/ET), premixed sealer was directly injected into the canal using a disposable syringe provided by the manufacturer; the F3 GP cones were deliberately inserted into the canal. The excess GP was cut at orifice level with a System B device.

The canal entrance then was filled with temporary restorative material (Caviton, GC Dental Industrial Corp., Tokyo, Japan), and the specimens were stored under 100% humidity at 37 °C for 7 days, to facilitate sealer setting.

### 2.3. Initial Micro-CT Scan Protocol

All teeth were scanned using a micro-CT system (SKYSCAN 1272, Bruker microCT, Kontich, Belgium) at 70 Kv, 142 µA, 0.5-mm aluminum filter, 180° rotation, 0.6° rotation step, and frame averaging of 4; this protocol produced a pixel size of 12 µm. To reconstruct the scanned digital images, Data Viewer 64 software (version 1.5.2.4, Bruker) was used, and CT-An software (version 1.16.1.0, Bruker) was used to analyze the volume of the intracanal void. All images were examined by a single blinded observer.

To analyze the three-dimensional (3D) volume of the void, binary images were used; the region of interest was selected on each cut to calculate the percentage of void from the canal. 3D analysis was performed in the whole canal; subgroup analyses were also performed separately at the apical (0–3 mm), middle (3–6 mm), and coronal (6–9 mm) levels.

Two-dimensional (2D) analysis was conducted to assess void occurrence. Cross-sectional images perpendicular to the long axis of the root were obtained; the images were transformed into binary images using image thresholding to verify the intracanal void. The distribution of the void was evaluated from the apex to the 9-mm level.

### 2.4. GP Removal Procedures and Second Micro-CT Scan Protocol

In all groups, the removal of obturation materials was performed using #4 and #3 Gates Glidden drills (Dentsply Maillefer) and ProTaper universal Re-treatment files (Dentsply Sirona). Then, a profile system (Dentsply Maillefer) of #35/.06 size was used. All instrumentation was performed using 2.5% sodium hypochlorite. All teeth were scanned using micro-CT with the same settings as the first scan; the remaining obturation materials were calculated with 3D analysis.

### 2.5. Re-Obturation and Final Micro-CT Scan Protocol

In the AP/AP and ET/AP groups, each canal was re-obturated with #35/.06 GP cone and AH Plus using CWT, as performed in the initial AP groups. In the AP/ET and ET/ET groups, each canal was re-obturated with a #35/.06 GP cone, and Endoseal TCS using SCT, similar to the approach used in the initial ET groups.

After sealer setting, all samples were scanned on the final micro-CT using the settings applied in the initial scans. 3D analysis was performed to analyze the percentage of void volume; 2D analysis was conducted to identify the distribution of the void in the whole canal and at the apical, middle, and coronal levels.

### 2.6. Statistical Analysis

The normality of the data distribution was examined using the Shapiro–Wilk test; it showed a normal distribution. Therefore, Student’s *t*-tests and a one-way analysis of variance were used to analyze the percentage of void volume, and the percentage of remnant materials. All statistical analyses were performed with SPSS software (version 25, SPSS Inc., Chicago, IL, USA). The significance level was established at *p* < 0.05.

## 3. Results

Table 1 shows the mean percentage volumes of voids after initial obturation by using AH plus and Endoseal TCS. In the whole canal and apical third, the Endoseal TCS groups showed a significantly smaller void volume than the AH plus groups did (*p* < 0.05). There were no significant differences between the sealer groups at the coronal and middle third (*p* > 0.05).

**Figure 1:**
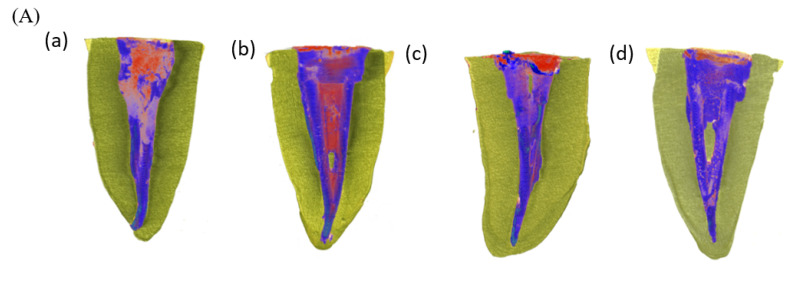

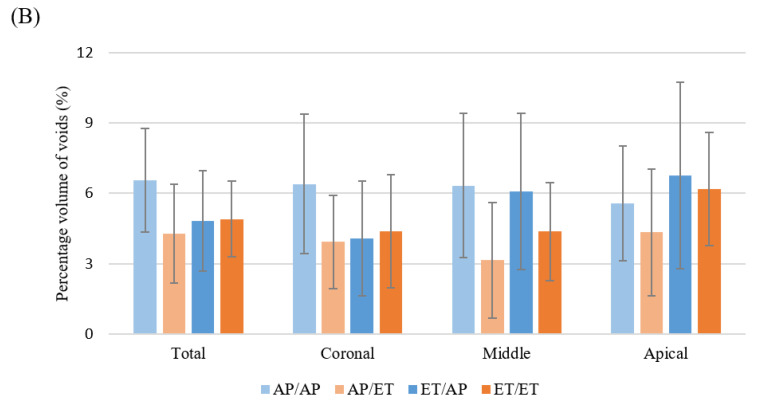
(**A**) Representative three-dimensional reconstructions after re-obturation. Filling materials (GP and sealer) are represented in blue, and voids are represented in red. (**a**) AP/AP; (**b**) AP/ET; (**c**) ET/AP; (**d**) ET/ET. (**B**) Percentage volume of voids (Mean ± standard deviation) after re-obturation in 4 groups in the whole canal and in the different thirds. There are no significant differences between the groups in the same portions (*p* > 0.05).

**Figure 2:**
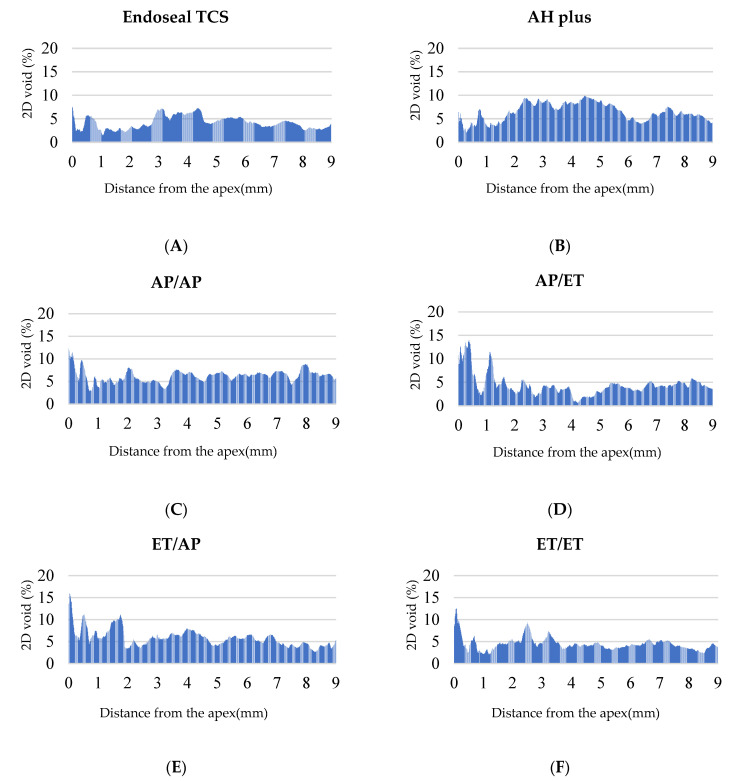
Two-dimensional analysis of void percentage along the distance from the apex. (**A**) Initial obturation of AH plus with CWT; (**B**) initial obturation of Endoseal TCS with SCT; (**C**) re-obturation of AP/AP group, (**D**) re-obturation of AP/ET group; (**E**) re-obturation of ET/AP group; (**F**) re-obturation of ET/ET group.

After GP removal, the Endoseal TCS group had a significantly greater percentage than did the AH Plus group (Endoseal TCS: 2.54 ± 1.56%, AH Plus: 1.74 ± 0.87%) (*p* < 0.05). Figure 1 shows the representative 3D images of the four groups after Re-treatment. There were no significant differences in filling quality among all groups after re-obturation (*p* > 0.05) (Figure 1). There were no significant differences when comparing the quality of Re-treatment in the groups that had used AH Plus and Endoseal TCS in the initial treatment (*p* > 0.05) (Table 2). With respect to the sealer used during Re-treatment, Endoseal TCS had a significantly smaller percentage volume of void compared with AH Plus, but only in the middle third (*p* < 0.05); there were no significant differences in the whole canal and other thirds, regardless of which sealer had been used for the initial treatment (*p* > 0.05) (Table 3). There were no significant differences in Re-treatment filling quality between the groups that used the same sealer and the groups that used different sealers for initial treatment and Re-treatment (*p* > 0.05) (Table 3). Initial treatment and Re-treatment had similar obturation quality (*p* > 0.05) (Table 4). Figure 2 shows the distribution of the void according to distance from the apex, after initial obturation and re-obturation.

## 4. Discussion

Proper void-free root canal filling is a key factor for successful nonsurgical endodontic treatment [18,19]. The role of the root canal sealer is to obturate the interfacial gaps between the root dentin and the filling core, and it should have excellent sealing property to minimize the formation of voids in the root canal [20]. In this study, micro-CT was used to evaluate the obturation quality after initial endodontic treatment and Re-treatment with two different sealers, and to identify intracanal residual materials after the removal procedure. Previous studies have demonstrated that this method can accurately quantify the filling materials and assess its quality without damaging the specimen [21,22]. In this study, the same specimen could be evaluated at various sequences of endodontic Re-treatment, unlike other existing evaluation methods.

In recent years, interest in endodontic Re-treatment has been increasing with the extension of the human lifespan and advancements in endodontics. Classically, AH Plus was considered a “gold standard” sealer because of its satisfactory properties for clinical usage; it has been widely used in endodontics [23,24,25]. However, calcium silicate-based sealers have recently been introduced and are gaining popularity. Therefore, teeth initially treated with AH Plus have sometimes been re-treated using calcium silicate-based sealer with the single-cone technique. To our knowledge, there have been no studies concerning the quality of obturation after endodontic Re-treatment. In this study, we investigated whether calcium silicate-based sealer could be a satisfactory treatment option with respect to obturation quality after the Re-treatment of teeth that initially had been treated with AH Plus.

In this study, micro-CT was used to evaluate obturation quality after the initial endodontic treatment and Re-treatment with two different sealers, then to identify intracanal residual materials after the removal procedure. Previous studies have demonstrated that this method can accurately quantify the filling material and allow quality assessment without damage to the specimen [21,22]. In this study, we therefore evaluated the same specimen during various steps of endodontic Re-treatment, in contrast to previous assessment methods.

In the initial root canal treatment, Endoseal TCS with SCT showed a lower void volume, compared with AH Plus with CWT, on the whole canal and the apical third (*p* < 0.05). Both showed satisfactory obturation quality. In a previous study, single-cone technique (SCT) showed inadequate obturation in the oval-shaped canals [26]. Some studies have shown that AH plus with CWT and calcium silicate-based sealer with SCT had comparable obturation quality in both single-rooted premolars and mesial canals of mandibular molars [12,13]. Since the oval-shaped canal was treated as two canals in the present study, it is speculated that similar or fewer voids were observed in the single-cone technique than in the continuous wave technique.

For both groups, the removal of obturation material was properly performed using Gates Glidden drills and Ni-Ti Re-treatment files. There were no specimens from which the obturation material was completely removed, although significantly greater quantities of residue were present in the Endoseal TCS groups than in the AH Plus groups. This finding is consistent with previous results, whereby more remaining materials were present when using the Endosequence BC sealer than when using the AH Plus sealer [27]. This is potentially because calcium silicate-based sealer adheres to root dentin [28]. Additionally, the tag-like structure of calcium and phosphate, which may reflect intratubular precipitation, might contribute to dentin bonding and sealing properties of calcium silicate-based sealers [29].

The final micro-CT scans showed the obturation quality of the endodontic Re-treatment. There were no significant differences among the four groups. Although the primary sealer showed differences concerning root canal filling retrievability, it did not affect the outcome of Re-treatment (Table 2). This supported an independent analysis of initial treatment and Re-treatment. In the Re-treatment, Endoseal TCS and AH Plus had similar obturation quality, regardless of the previously used sealer, except in the middle third (*p* = 0.03). Therefore, the null hypothesis was accepted. The middle third was presumably most affected by the residue from the previous step: this difference was unexpected. The use of different sealers for the initial treatment and Re-treatment did not lead to a significant difference, compared with the use of the same sealer for both treatments. This suggests that both AH Plus and Endoseal TCS could be selected for Re-treatment of teeth to which AH Plus was initially applied.

Endodontic Re-treatment poses biological and technical challenges [30]. In a previous retrospective study, 95% of re-treated teeth showed acceptable homogeneity according to assessments of patient radiographs [31]. However, no study has used micro-CT to compare 3D filling quality between primary endodontic treatment and Re-treatment. The present research showed that there were no significant differences in obturation quality between primary and secondary endodontic treatments (*p* > 0.05). However, 2D analysis showed a tendency for a greater proportion of voids at 1 mm near the apex after Re-treatment, compared with the initial treatment (Figure 2). This is presumably because it is impossible to entirely remove the original obturation materials and re-obturate the new filling core up to the apical foramen during endodontic Re-treatment. This is a limitation of root canal Re-treatment, and may explain the lower success rates during Re-treatment, compared with primary endodontic treatment in previous studies [32,33].

One of the limitations of the current study was that it only evaluated filling quality in single-rooted teeth with oval-shaped canals. The difficulty and integrity of root canal obturation are influenced by the anatomic structures of the canal. Therefore, additional studies are needed to investigate these factors in teeth with greater curvature or more complex microstructures.

Another limitation is that we utilized micro-CT analysis. This could have been limiting as the object of interest could be affected; the analyst must establish the region of interest, and the threshold value is set by the analyst. Nevertheless, micro-CT was adopted in this study because of the many advantages described above. To evaluate the other factors of success of endodontic Re-treatment, further physicochemical analyses and additional biologic studies must be needed.

## 5. Conclusions

Endoseal TCS sealer with SCT and AH Plus sealer with CWT showed satisfactory obturation quality during both initial endodontic treatment and endodontic Re-treatment. It also showed similar filling quality, regardless of whether the same sealers or different sealers were used for the first treatment and Re-treatment. Therefore, both Endoseal TCS sealer and AH Plus sealer could be appropriate choices for endodontic Re-treatment, regardless of which sealer was used in the previous treatment.

## Figures and Tables

**Table 1:** Percentage volumes of voids (mean ± standard deviation) after first obturation using AH plus and Endoseal TCS in the whole canal and in each third.

Group/Region	Percentage Volume of Voids (%)	
	AH Plus (AP/AP + AP/ET)	Endoseal TCS (ET/AP + ET/ET)
Total	5.08 ± 2.07 *	3.75 ± 1.89 *
Coronal	4.69 ± 2.31	3.59 ± 2.11
Middle	6.01 ± 3.57	4.25 ± 3.95
Apical	5.54 ± 3.85 *	3.09 ± 3.49 *

* There are statistically significant differences between the sealer groups in the same portions (*p* < 0.05).

**Table 2:** Percentage volumes of voids (mean ± standard deviation) after re-obturation according to the first used sealer in the whole canal and in each third.

Group/Region	Percentage Volume of Voids (%)	
	AH Plus (AP/AP + AP/ET)	Endoseal TCS (ET/AP + ET/ET)
Total	5.41 ± 2.40	4.86 ± 1.84
Coronal	5.16 ± 2.76	4.23 ± 2.37
Middle	4.74 ± 3.17	5.22 ± 4.03
Apical	4.95 ± 2.59	6.46 ± 3.21

There are no significant differences between the groups in the same portions (*p* > 0.05).

**Table 3:** Percentage volumes of voids (mean ± standard deviation) after re-obturation using AH Plus and Endoseal TCS, regardless of the previous sealer, and after re-obturation using the same or different sealers for initial treatment and Re-treatment in the whole canal and each third.

Group /Region	Percentage Volume of Voids (%)			
	Re-treatment with AH Plus (AP/AP + ET/AP)	Re-treatment with Endoseal TCS (AP/ET + ET/ET)	Same Sealer (AP/AP + ET/ET)	Different Sealer (ET/AP + AP/ET)
Total	5.69 ± 2.29	4.59 ± 1.86	5.73 ± 2.06	4.54 ± 2.08
Coronal	5.23 ± 2.90	4.15 ± 2.16	5.38 ± 2.83	4.00 ± 2.16
Middle	6.21 ± 4.23 *	3.76 ± 2.32 *	5.35 ± 2.76	4.61 ± 4.31
Apical	6.15 ± 3.27	5.25 ± 2.66	5.86 ± 2.39	5.54 ± 3.53

* There are statistically significant differences. (*p* < 0.05).

**Table 4:** Percentage volumes of voids (mean ± standard deviation) after obturation during initial treatment and Re-treatment in the whole canal and in each third.

Group/Region	Percentage Volume of Voids (%)	
	Initial Treatment	Re-treatment
Total	4.41 ± 2.07	5.14 ± 2.13
Coronal	4.14 ± 2.25	4.69 ± 2.58
Middle	3.76 ± 2.32	6.21 ± >4.23
Apical	5.25 ± 2.66	6.15 ± 3.27

There are no significant differences between the groups in the same portions (*p* > 0.05).

## Data Availability

Not applicable.
